# Influence of microbial cell morphology and composition on radio frequency heating of simple media at different frequencies

**DOI:** 10.1038/s41598-023-35705-4

**Published:** 2023-07-05

**Authors:** Julian Espitia, Davy Verheyen, Dmytro S. Kozak, Jan F. M. Van Impe

**Affiliations:** 1 BioTeC+-Chemical and Biochemical Process Technology and Control, KU Leuven, Gebroeders de Smetstraat 1, 9000 Gent, Belgium; 2 Physico-Technological Institute of Metals and Alloys of the National Academy of Sciences of Ukraine, 34/1 Acad. Vernadskogo Boul., Kiev, 03142 Ukraine

**Keywords:** Applied microbiology, Pathogens

## Abstract

The effect of *Listeria monocytogenes*, *Salmonella* Typhimurium, and *Saccharomyces cerevisiae* on RF heating was studied in sterilized Milli-Q water and saline solution during treatments at 27.0 ± 0.6 MHz and 3.0 ± 0.02 MHz for 30 min. The presence of microorganisms caused a significant increase in temperature (maximum to 54.9 °C), with no significant decrease in cell numbers being observed for any conditions. For both media and frequencies, heating rates followed the order *S.* Typhimurium ≤ *L. monocytogenes* ≤ *S. cerevisiae*, except for heating at 3.0 ± 0.02 MHz in saline solution, where heating rates for *S. cerevisiae* and *S*. Typhimurium were equal. Generally, heating rates for microorganisms were significantly higher at 27.0 ± 0.6 MHz than at 3.0 ± 0.02 MHz, except for the *S. cerevisiae* case. Observed phenomena were probably caused by differences in the cell lipid and peptidoglycan content, with interaction effects with salt being present. This study was the first to investigate the influence of the presence of microorganisms on heating behavior of simple media. On the long term, more research on this topic could lead to finding specific RF frequencies more suitable for the heating of specific media and products for various applications.

## Introduction

Radio Frequency (RF) is a type of electromagnetic radiation with frequencies ranging from 3 kHz to 300 MHz. RF is a fast and volumetric heating technology in which molecular friction caused by ionic conduction and dipole rotation generates heat inside dielectric materials^1^. RF treatments drive moisture from the inside to the outside of products, avoiding overheating and over-dehydration of the product surface, since the interior heats faster than the surface^2^. Therefore, RF as a heating technique has been already highly applied in the food industry, for processes such as thawing^3–5^, drying^6,7^, disinfestation^8–10^, and inactivation of enzymes^11,12^. Furthermore, RF treatments have also shown to be an effective technique for food pasteurization^13–15^, enabling the inactivation of pathogenic microorganisms in different food products^16,17^. Hence, RF constitutes a promising and important modern heating technique in the food processing industry^18^.

In the food industry, heating is often applied in relation to the presence of microorganisms. On the one hand, microorganisms play an important positive role in the food industry, from traditional industrial biotechnology applications such as dairy products, wine, and beer production, and fermented foods, to industrial fermentation dedicated to the manufacturing of solvents, organic acids, vitamins, enzymes, and other products^19^. On the other hand, many microorganisms can also cause diseases when present in food products^20^. Therefore, heating of microorganisms in the food industry can have two purposes, i.e., (i) providing an optimal environment for microorganisms to grow and serve their positive role, or (ii) inactivating harmful microorganisms to ensure food safety. Concerning heating techniques to provide an optimal environment for microorganisms, two notable examples are increasing the temperature to enable fermentation in yoghurt and beer brewing, where with increasing fermentation temperature, the cycle time and production capacity can be shortened^21^. Concerning heating techniques for microorganism inactivation in foods, the most common process is pasteurization, which is accepted as an effective preservation method to inactivate dangerous amounts of pathogens present in food products without compromising desirable food quality^22^.

RF treatments are widely applied on microorganisms for pasteurization in several foods^23^ and agricultural products^24^. Most of the time, the heating of the foods, due to the dielectric losses inherent to electromagnetic exposure, is regarded as the dominant cause of microbial inactivation during pasteurization^25^. Nevertheless, the existence of lethal or sublethal nonthermal effects of dielectric heating (e.g., RF, microwave) on microorganisms is a possibility, although their existence is heavily discussed^26–28^. Such nonthermal effects could encompass (i) microbial inactivation occurring below typical inactivation temperatures (e.g., 50 °C) and (ii) microbial inactivation mechanisms at typical inactivation temperatures, but different as compared to purely thermal inactivation. In relation to this, the effect of RF on microorganisms has been studied to some extent, with some studies having shown the effect of RF treatments on bacterial cell membranes^29^, as well as the non-thermal effect at certain frequencies^16,30^. Nevertheless, the opposite case, i.e., the effect that cells might cause to the heating process is, to the best knowledge of the authors, still not fully established. This type of knowledge, in combination with the already available knowledge concerning the influence of certain food/media components (e.g., salt, fat) on heating behavior^31^, would be especially valuable to design more effective heating processes in the food industry, for instance enabling the selection of a suitable RF frequency to acquire required temperature profiles for specific food processes, e.g., fermentation. This is due to the fact that RF heating behavior is for a large part determined by the dielectric properties of the product, i.e., the dielectric constant ε′, expressing the ability of a material to store electrical energy, and the loss factor ε″, expressing the ability to dissipate the energy as heat. Dielectric properties are dependent on a plethora of factors, such as moisture content, chemical composition (e.g., fat, protein, carbohydrates, salt), structure, temperature, and the applied frequency^32^. Each specific product could hence have a specific RF frequency at which it could be treated more effectively. In this regard, RF frequencies of 13.56, 27.12, and 40.68 MHz are currently allowed for industrial, scientific and medical applications^32^. Therefore, these currently allowed frequencies have the most immediate application potential. On the long term, finding more suitable RF frequencies (e.g., within the 1–30 MHz range) for microorganism heating (and inactivation) could showcase the need for additional allowed frequency bands for industrial applications.

In the current study, the aim is to acquire an initial view on the effect of microorganisms on the RF heating behavior of simple media, dependent on (i) microbial cell morphology and composition, (ii) RF frequency, and (iii) medium composition. The effect of each of the three aforementioned influencing factors is taken into account. It is assumed that, if the presence of microorganisms affects RF heating behavior, the microbial cells absorb a certain amount of energy when being exposed to RF, in turn causing a rise in the internal temperature of the medium at the location of the microorganisms. This should hence lead to the observation of different RF heating rates when microorganisms are present in the medium. During such experiments, any possible nonthermal effects on the microorganism can also be identified. Concerning microorganisms, high concentrations of *Listeria monocytogenes* (i.e., Gram-positive bacterium), *Salmonella* Typhimurium (i.e., Gram-negative bacterium), and *Saccharomyces cerevisiae* (i.e., yeast) were selected. In order to investigate the effect of RF frequency, two frequencies were selected, i.e., 27.0 ± 0.6 MHz and 3.0 ± 0.02 MHz. On the one hand, the 27.0 ± 0.6 MHz frequency encloses the 27.12 MHz frequency which is used most in industry, providing a suitable benchmark case. On the other hand, the 3.0 ± 0.02 MHz frequency, while not currently allowed for industrial, scientific and medical applications, provides a frequency of one order of magnitude lower than the first frequency, enabling to clearly distinguish general trends on the effects of RF frequency on heating behavior. In future research, the acquired knowledge can be used to select a suitable allowed frequency for specific heating applications. For the medium composition, Milli-Q water (MQW) and saline solution (i.e., NaCl in distilled water) were selected, as salt is one of the most important influencing factors on RF heating due to the ionic depolarization occurring during the process. In these simple media, not containing a high number of components that influence RF heating due to their influence on dielectric properties, it is more straightforward to identify possibly small effects of microorganisms on the RF heating behavior. To the best knowledge of the authors, the present study is the first to investigate the effect of the presence of microorganisms on the heating behavior of simple media.

## Methods

### Description of experiments

RF thermal treatments were performed for the three microorganisms (*L. monocytogenes*, *S*. Typhimurium, and *S. cerevisiae*) in MQW and NaCl medium. A total of 8 experiments per frequency (27.0 ± 0.6 MHz and 3.0 ± 0.2 MHz) were performed. Each experiment consisted of 10 replicates of 3 mL volume per medium (i.e., without microorganisms) and medium-microorganism combination.

### Microorganisms and culture conditions

*Listeria monocytogenes* LMG 23774 and *Salmonella* Typhimurium LMG 14933 were acquired from the BCCM/LMG bacteria collection of Ghent University in Belgium, and *Saccharomyces cerevisiae* strain S288c ATCC 204508 was acquired from the ATCC collection. Brain Heart Infusion Broth (BHI, VWR International, Leuven, Belgium), Tryptic Soy Broth (TSB, VWR International, Leuven Belgium), and Yeast Extract Peptone dextrose (YPD, Carl Roth GmbH, Germany) were used as liquid culture media for *L. monocytogenes*, *S*. Typhimurium, and *S. cerevisiae,* respectively. Stock cultures were stored at – 80 °C in 80% (v/v) of the respective nutrient broth and 20% (v/v) glycerol (Acros Organics, Geel, Belgium) for each bacterium, and 75% (v/v) of YPD medium and 25% (v/v) glycerol (Acros Organics, Geel, Belgium) for yeast. Purity plates for each microorganism were prepared by using a loop and spreading stock culture onto agar plates, which were prepared by the addition of 1.4% (w/v) bacteriological agar (VWR International, Leuven, Belgium) to the respective nutrient broth. Purity plates were stored for 24 h at 30 °C for *L. monocytogenes* and *S. cerevisiae,* and at 37 °C for *S.* Typhimurium. For each bacterium, one colony from the purity plate was transferred into an Erlenmeyer flask containing 20 mL of the respective nutrient broth. The Erlenmeyer flasks were incubated for 24 h at 30 or 37 °C *for L. monocytogenes* and S. *Typhimurium*, respectively. Afterwards, 20 µL of broth was transferred to a fresh nutrient broth Erlenmeyer flask and incubated under the same conditions, resulting in stationary phase cultures with a cell density of approximately 10^8^ CFU/mL. For *S. cerevisiae*, two colonies were transferred from the purity plate into an Erlenmeyer flask containing 20 mL of YPD broth and stored for 24 h at 30 °C while shaking at 220 rpm, resulting in stationary phase cultures with a cell density of approximately 10^7^ CFU/mL. This lower cell density for yeast cells was selected to counteract the bigger size of the yeast cells as compared to bacterial cells.

### Inoculation in liquid media

Sterilized Milli-Q water (distilled water deionized to a resistivity of 18.2 MW cm at 25 °C, Millipore) (MQW) and 0.9% NaCl solution (NaCl, Merck Life Science BV, Hoeilaart, Belgium) were selected as heating media. For each microorganism, 9 mL of the second preculture was transferred to a 50 mL conical centrifuge tube and centrifuged at 18,500*g* for 15 min at 4 °C. Afterwards, the supernatant was carefully removed, and the cells were washed twice using sterilized Milli-Q water and centrifuging under the same conditions. The remaining pellet of cells was collected by adding a small volume of the medium (MQW or NaCl), strongly mixing with an inoculation loop, and afterwards transferring the mixture back to a total media volume of 30 g. Afterwards, the inoculated media were thoroughly mixed and then divided over small glass vials (4 mL, 45 × 14.7 mm, BGB Analytic Benelux B.V., Harderwijk, the Netherlands) which were stored at 10 °C for approximately 15 h before the RF experiments. Each vial contained 3 mL of inoculated MQW or NaCl.

### Radiofrequency thermal experiments

RF experiments were conducted using two small-scale laboratory RF setups, built according to the flyback topology design using an air-core transformer (Tesla coil), as described for an 27.12 MHz setup in Kozak et al*.*^33^. Both RF set-ups were free-running oscillators operated at 27.0 ± 0.6 MHz and 3.0 ± 0.02 MHz, respectively. The power consumption of treated samples was approximately 7.0 ± 0.6 W (3–4 W without sample load). For both setups, a single electrode, upon which samples were placed, was used. For the setup operating at 27.0 ± 0.6 MHz, this was a copper disk with a diameter of 16.25 mm. For the 3.0 ± 0.02 MHz setup, the required frequency was obtained by using a copper disk with a diameter of 21 mm. A step-up transformer with 675 turns for the secondary coil was used, wound on a 15 mm diameter glass tube. A variable capacitor with capacitances from 25 to 150 pF was connected in parallel to the primary coil having 4 turns, and to the emitter of the NPN transistor (KT805AM). The other parts of both RF setups (e.g., power supply, cooling fan) were constructed as described in detail in Kozak et al*.*^33^. All experiments were conducted using 3 mL samples in the mentioned small glass vials, which were equipped with a polypropylene screw cap lid with a glass capillary tube inserted through the middle of the lid (i.e., to enable temperature measurements). Samples were placed in a polystyrene holder with the same height and diameter as the glass vial, in order to limit heat dissipation to the environment. The temperature profile of samples was recorded by using a TS3 GaAs-based optic fiber temperature sensor, placed in the sample through the glass capillary, connected to a Bench Top FOTEMP-PLUS signal conditioner (Weidmann Technologies Deutschland GmbH, Dresden, Germany) and data was processed using the FOTEMP-Assistant software (Optocon, AG, Dresden, Germany). Before RF treatment, samples were placed at 20 ºC in order to start at the same temperature. A treatment time of 30 min was selected, since preliminary experiments showed that temperature increases became negligible at longer treatment times, and the objective was to investigate the effect on the heating rate. During the experiments, the sample temperature was recorded every second.

### Microbiological quantification

Treated samples were placed in cold water (from 14 to 16 °C) in order to stop possible inactivation, before being stored at 4 °C prior to further sample processing. Afterwards, serial dilutions from 10^–1^ to 10^–6^ were made using 0.9% NaCl solution. The final density of cells was quantified via the drop method^34^, i.e., three 20 μL drops per dilution were placed on BHI, TSA, and YPD agar for *L. monocytogenes*, *S.* Typhimurium, and *S. cerevisiae*, respectively.

### Statistical analysis

Analysis of variance (ANOVA, single variance) test at 95.0% confidence level (α = 0.05) was performed in order to find significant differences among the inoculated and non-inoculated media. Fisher’s Least Significant Difference test was used to distinguish which means were significantly different from others. Standardized skewness and standardized kurtosis were used to evaluate if datasets come from normal distributions. The analyses were performed using MATLAB version R2020b (TheMathworks Inc., Natick, USA). Test statistics were regarded as significant when P ≤ 0.05.

## Results

### RF inactivation

Table 1 provides an overview of the cell density for the medium-microorganism combinations at 27.0 ± 0.6 and 3.0 ± 0.02 MHz (the complete dataset for all microorganisms is available in Supplementary Data S1 online). *L. monocytogenes* and *S*. Typhimurium reached an initial density (*N*_*o*_) of 10^8^ CFU/mL after 48 h pre-culture and *S. cerevisiae* had an initial density of 10^7^ CFU/mL after 24 h pre-culture, which was the highest reachable density for each microorganism. After 30 min treatment, statistical differences between *N*_*0*_ and the cell density after treatment (*N*_*af*_) were observed for *S. cerevisiae* in MQW for both frequencies and in NaCl medium at 3.0 ± 0.02 MHz. Also, *S.* Typhimurium showed statistical differences in NaCl for both frequencies. Nevertheless, this cannot be attributed to an inactivation due to the small reduction within *N*_*0*_ and *N*_*af*_. Therefore, these significant differences can be taken as negligible.

**Table 1:** Average cell density and standard deviation of each medium-microorganism combination for RF treatments at 27.5 ± 0.6 MHz and 3.0 ± 0.02 MHz.

Medium-microorganism combinations	Setup 27.0 ± 0.6 MHz		Setup 3.0 ± 0.02 MHz	
	*N*_*o*_	*N*_*af*_	*N*_*o*_	*N*_*af*_
MQW				
MQW-*L. monocytogenes*	8.64 ± 0.07^a^	8.63 ± 0.11^a^	8.66 ± 0.03^a^	8.61 ± 0.15^a^
MQW-*S. Typhimurium*	8.68 ± 0.03^a^	8.62 ± 0.14^a^	8.68 ± 0.03^a^	8.62 ± 0.06^a^
MQW-*S. cerevisiae*	7.71 ± 0.08^a^	7.55 ± 0.05^b^	7.71 ± 0.08^a^	7.59 ± 0.05^b^
NaCl				
NaCl-*L. monocytogenes*	8.69 ± 0.01^a^	8.59 ± 0.08^a^	8.69 ± 0.01^a^	8.59 ± 0.07^a^
NaCl-*S. Typhimurium*	8.68 ± 0.03^a^	8.58 ± 0.05^b^	8.68 ± 0.03^a^	8.59 ± 0.04^b^
NaCl-*S. cerevisiae*	7.77 ± 0.10^a^	7.67 ± 0.05^a^	7.77 ± 0.10^a^	7.63 ± 0.05^b^

Initial cell density (*N*_*0*_), and density after treatment (*N*_*af*_) are expressed in log (CFU/mL). Different lowercase letters indicate significant differences in *N*_*0*_ and *N*_*af*_ for one medium-microorganism combination at one frequency (P ≤ 0.05). At least two independent experiments were conducted for each condition (plate count data for each condition is available in Supplementary Data  S1 online).

### RF treatment effect on cells

**Figure 1:**
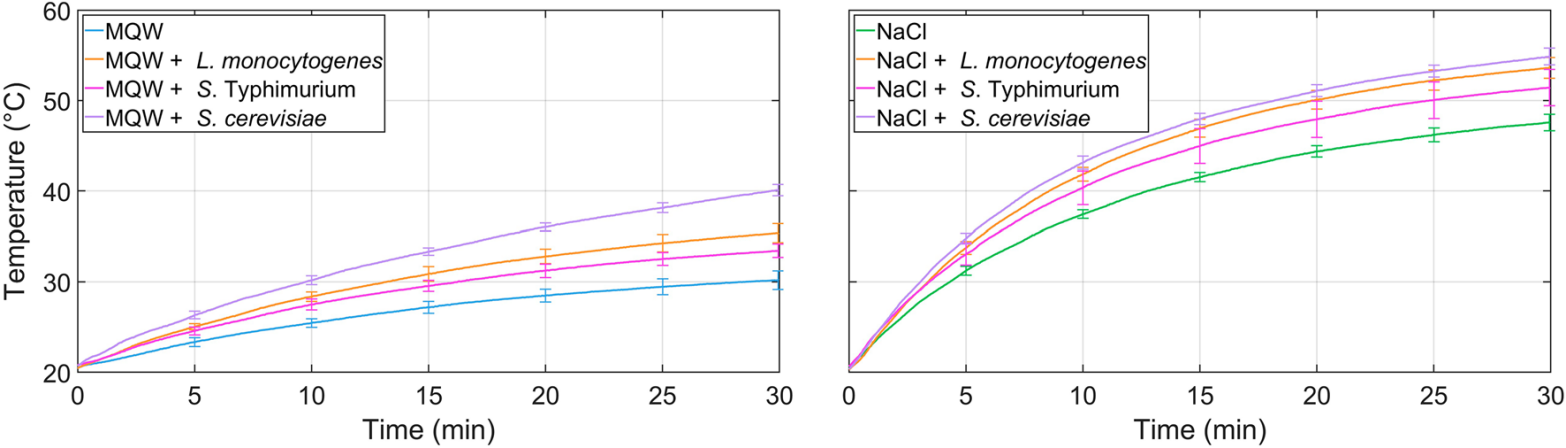
Average temperature profiles and standard deviation (every 5 min) for *L. monocytogenes*, *S.* Typhimurium, and *S. cerevisiae* at 27.0 ± 0.6 MHz. Ten independent experiments were conducted for each condition.

**Figure 2:**
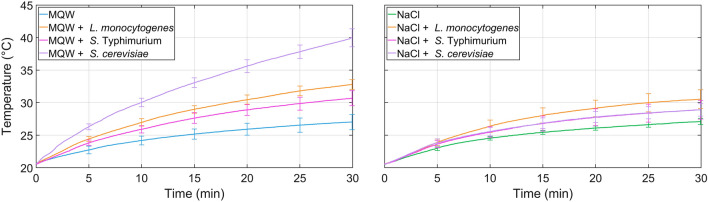
Average temperature profiles and standard deviation (every 5 min) for *L. monocytogenes*, *S.* Typhimurium, and *S. cerevisiae* at 3.0 ± 0.02 MHz. Ten independent experiments were conducted for each condition.

Figures 1 and 2 illustrate the average temperature profiles and standard deviation every 5 min of the 10 experiments for each microorganism at 27.0 ± 0.6 and 3.0 ± 0.02 MHz, respectively (the complete dataset of the temperature profiles for each condition and statistical analysis is available in Supplementary Data S2 online).

First of all, a statistical analysis was performed every 5 min to compare the temperature of microorganisms within the same medium. MQW temperature was significantly lower than any MQW-microorganism combination during the whole treatment for both frequencies. With the exception of the first 5 min of treatment (no significant differences between *L. monocytogenes* and *S*. Typhimurium), MQW-microorganisms combination temperature increases followed the order *S*. Typhimurium < *L. monocytogenes* < *S. cerevisiae*. NaCl media temperatures were significantly lower than all NaCl medium-microorganism combination during the whole treatment for both frequencies. At 27.0 ± 0.6 MHz, starting from 10 min of treatment, NaCl-microorganisms combination temperature increases followed the order *S*. Typhimurium < *L. monocytogenes* = *S. cerevisiae*, while no statistical differences among the two bacteria were observed after 5 min. At 3.0 ± 0.02 MHz, starting from 20 min of treatment, NaCl-microorganisms combination temperature increases followed the order *S*. Typhimurium = *S. cerevisiae* < *L. monocytogenes*, while limited statistical differences among microorganisms were observed after 5 and 10 min.

Secondly, a statistical analysis was performed to determine differences within the same medium or medium-microorganisms combination and different frequencies. Temperatures of MQW and MQW-bacteria combinations (*L. monocytogenes* and *S.* Typhimurium) were consistently higher at 27.0 ± 0.6 MHz than at 3.0 ± 0.02 MHz. *S. cerevisiae*, on the contrary, did not showcase any statistical difference in temperatures among frequencies in MQW media. NaCl media showed significant differences among both frequencies in all media and medium-microorganism combinations. Higher temperatures were always present at 27.0 ± 0.6 MHz than at 3.0 ± 0.02 MHz.

Heating rate (°C/min) and standard deviations were also calculated at the intervals 0–5, 5–10, 10–15, 15–20, 20–25, and 25–30 min for each experiment. Statistical analyses of heating rates for medium and medium-microorganism combinations at two frequencies (27.5 ± 0.6 MHz and 3.0 ± 0.02 MHz) are provided in Table 2.

**Table 2:** Heating rate (ºC/min) and standard deviation, for 5 min intervals, of experiments at 27.0 ± 0.6 and 3.0 ± 0.02 MHz.

Heating rate (ºC/min) for 27.0 ± 0.6 MHz set-up								
Time intervals (min)	MQW media				NaCl media			
	MQW	*L. monocytogenes*	*S.* Typhimurium	*S. cerevisiae*	NaCl	*L. monocytogenes*	*S. Typhimurium*	*S. cerevisiae*
0–5	0.56 ± 0.08^a,B^	0.91 ± 0.10^c,B^	0.76 ± 0.08^b,B^	1.12 ± 0.07^d,A^	2.13 ± 0.11^a,B^	2.69 ± 0.18^bc,B^	2.48 ± 0.28^b,B^	2.88 ± 0.11^c,B^
5–10	0.43 ± 0.05^a,B^	0.67 ± 0.05^c,B^	0.58 ± 0.04^b,B^	0.77 ± 0.04^d,A^	1.25 ± 0.04^a,B^	1.63 ± 0.10^c,B^	1.46 ± 0.15^b,B^	1.68 ± 0.09^c,B^
10–15	0.34 ± 0.05^a,B^	0.50 ± 0.07^c,B^	0.41 ± 0.04^b,B^	0.63 ± 0.02^d,A^	0.81 ± 0.03^a,B^	1.01 ± 0.08^c,B^	0.92 ± 0.05^b,B^	0.96 ± 0.04^cb,B^
15–20	0.26 ± 0.05^a,B^	0.39 ± 0.01^c,B^	0.34 ± 0.04^b,B^	0.54 ± 0.02^d,A^	0.57 ± 0.03^a,B^	0.63 ± 0.04^b,B^	0.59 ± 0.03^b,B^	0.63 ± 0.04^b,B^
20–25	0.20 ± 0.04^a,B^	0.29 ± 0.04^b,A^	0.26 ± 0.03^b,B^	0.42 ± 0.04^c,A^	0.37 ± 0.04^a,B^	0.44 ± 0.03^b,B^	0.42 ± 0.02^b,B^	0.43 ± 0.04^b,B^
25–30	0.15 ± 0.03^a,B^	0.23 ± 0.03^c,A^	0.18 ± 0.02^b,A^	0.39 ± 0.03^d,A^	0.28 ± 0.03^ab,B^	0.27 ± 0.03^a,B^	0.28 ± 0.03^ab,B^	0.32 ± 0.06^ab,B^

Heating rate (ºC/min) for 3.0 ± 0.02 MHz set-up								
Time intervals (min)	MQW media				NaCl media			
	MQW	*L. monocytogenes*	*S. typhimurium*	*S. cerevisiae*	*NaCl*	*L. monocytogenes*	*S. typhimurium*	*S. cerevisiae*
0–5	0.42 ± 0.08^a,A^	0.76 ± 0.07^b,A^	0.66 ± 0.09^b,A^	1.14 ± 0.10^c,A^	0.50 ± 0.05^a,A^	0.66 ± 0.11^b,A^	0.61 ± 0.12^ab,A^	0.66 ± 0.11^b,A^
5–10	0.29 ± 0.06^a,A^	0.51 ± 0.05^c,A^	0.42 ± 0.04^b,A^	0.75 ± 0.05^d,A^	0.31 ± 0.04^a,A^	0.50 ± 0.12^b,A^	0.37 ± 0.06^a,A^	0.35 ± 0.02^a,A^
10–15	0.20 ± 0.03^a,A^	0.40 ± 0.03^c,A^	0.34 ± 0.05^b,A^	0.60 ± 0.05^b,A^	0.18 ± 0.02^a,A^	0.33 ± 0.05^c,A^	0.26 ± 0.07^b,A^	0.26 ± 0.05^b,A^
15–20	0.15 ± 0.03^a,A^	0.30 ± 0.03^c,A^	0.25 ± 0.01^b,A^	0.51 ± 0.06^d,A^	0.13 ± 0.03^a,A^	0.22 ± 0.04^b,A^	0.19 ± 0.04^b,A^	0.19 ± 0.02^b,A^
20–25	0.13 ± 0.04^a,A^	0.27 ± 0.03^c,A^	0.20 ± 0.04^b,A^	0.44 ± 0.04^d,A^	0.11 ± 0.02^a,A^	0.17 ± 0.06^b,A^	0.13 ± 0.03^ab,A^	0.13 ± 0.02^ab,A^
25–30	0.09 ± 0.02^a,A^	0.21 ± 0.02^b,A^	0.16 ± 0.03^b,A^	0.44 ± 0.10^c,A^	0.09 ± 0.03^a,A^	0.11 ± 0.02^a,A^	0.10 ± 0.02^a,A^	0.09 ± 0.01^a,A^

Different lowercase letters indicate significant differences in temperature among microorganisms at the same frequency and medium (P ≤ 0.05). Different uppercase letters indicate significant differences in temperature among frequencies of one microorganism in one medium (P ≤ 0.05). Ten independent experiments were conducted for each condition (the complete dataset for the temperature profiles is available in Supplementary Data S2 online).

MQW heating rate was significantly lower than any MQW-microorganism combination during the whole treatment for both frequencies. MQW-microorganisms combination heating rates followed the order *S*. Typhimurium < *L. monocytogenes* < *S. cerevisiae* with the exception of 20–25 min and 25–30 min intervals, where *L. monocytogenes* and *S*. Typhimurium did not have significant differences in their heating rates at 27.5 ± 0.6 MHz and 3.0 ± 0.02 MHz, respectively.

NaCl heating rate was significantly lower than any NaCl-microorganism combination during almost all time intervals at 27.5 ± 0.6 MHz, with the exception of 25–30 min, where NaCl did not have significant differences with any NaCl-microorganism combination. At 3.0 ± 0.02 MHz, heating rate of NaCl-microorganism combinations were significantly higher than NaCl at 5–10 min. However, heating rates of NaCl and NaCl-microorganism combinations did not show any statistical differences at the end of the treatment (interval 25–30 min). Generally, heating rates for microorganisms were higher at 27.0 ± 0.6 MHz than at 3.0 ± 0.02 MHz, with the exception of *S. cerevisiae*, as heating rates did not show statistical differences within both frequencies.

## Discussion

### Thermal vs. nonthermal effects

Radiofrequency (RF) treatments offer a potential method for controlling microorganisms in agricultural commodities, for stimulating growth and metabolic production, on the one hand, and for pathogen inactivation, on the other^35^. Concerning RF inactivation, a distinction can be made between purely thermal and nonthermal inactivation. *L. monocytogenes*, *S.* Typhimurium, and *S. cerevisiae* have been successfully inactivated before by using different heat-inducing RF treatments^14,36,37^. However, in the present research, there was no significant decrease in the final cell density of any microorganism after 30 min of treatment. Highest internal temperatures for samples containing *L. monocytogenes* (55.4 °C), *S.* Typhimurium (54.1 °C) and *S. cerevisiae* (56.6 °C) were obtained in NaCl medium at 27.0 ± 0.6 MHz. At this RF frequency, studies have already confirmed the thermal inactivation of *L. monocytogenes* and *S*. Typhimurium at temperatures of 65 °C and higher^8,38,39^, and the thermal inactivation of *S. cerevisiae* at temperatures of 57 °C and higher^37^. Consequently, the sample temperatures obtained during 30 min of treatment in this study were probably not high enough to reduce the cell density. This evidences that the pasteurization process at 27.12 MHz is only related to a thermal effect^25,26^. While not studied previously, the results of the current study indicate a similar phenomenon at 3.0 ± 0.02 MHz. While there are studies that have proven the nonthermal inactivation of bacteria and yeasts by RF treatments^16,30^, this was at lower frequencies and different conditions than in the present study. Therefore, future studies on identifying possible nonthermal effects of electromagnetic energy on microorganisms should focus on different RF frequencies (e.g., lower than 3.0 MHz, higher than 27.0 MHz), or on longer exposure times at sublethal temperatures at the two used frequencies. In addition to purely nonthermal effects, it is also possible that inactivation mechanisms of thermal RF treatments (i.e., at temperatures above 70 °C) differ from traditional thermal treatments. To identify such effects, RF treatments should be conducted together with conventional thermal treatments, whereby the RF treatment temperature profiles should be mimicked as closely as possible in purely thermal inactivation experiments (i.e., to identify differences among RF and purely thermal inactivation). Finally, it should be taken into account that temperature changes resulting from RF exposure also lead to a change in the impedance value and dielectric properties of the food medium, necessitating an accurate control of the RF frequency which is dependent on the food dielectric properties.

### Factors affecting RF heating behavior

Differences in the heating rate of all experiments have shown that within the intervals from 0 to 20 min, there is a tendency of faster heating for microorganisms-medium combinations. As mentioned, the present study is, to the best knowledge of the authors, the first to systematically investigate this phenomenon. When microorganisms are exposed to an electromagnetic field, their membranes become charged^40^, allowing depolarization under the RF electromagnetic field and consequent heating. There are a number of factors that affect the overall efficiency of radio frequency heating, including radiation frequency, food composition, material size, salt content, moisture content, temperature, and density^35^. A variety of macronutrients affect dielectric and thermal properties of foods, e.g., proteins and carbohydrates are either dielectrically active or inert, while fat, ash, and moisture are often considered to exert the most consistent influence on dielectric properties^31^. Thus, the evidenced influence of microorganisms on the heating process by RF treatments in the present study might be related to the presence of the aforementioned compounds in each type of microorganism. Table 3 summarizes some of the main characteristics of microorganisms that may affect RF heating for each bacterium and yeast, i.e., size, shape, cell wall properties, and lipid content. In addition, the interaction of microorganisms with salt present in the heating medium also exerts an important influence. The possible influence of each mentioned factor is discussed in more detail in the paragraphs below. Hereby, it should be noted that the discussion is mainly based on the time intervals in which significant differences in heating rates among the different medium-microorganisms combinations were observed. At the later time intervals, heating rates were not sufficiently high (i.e., an attribute of the specific equilibrium between the applied RF power-time profile and occurring heat dissipation in the present study) to observe these significant differences.

**Table 3:** Characteristics and compounds present in the cells of *Salmonella*, *Listeria*, and *S. cerevisiae*.

	*Salmonella* (Gram-negative)	*Listeria* (Gram-positive)	*S. cerevisiae* (Eukaryote)
Size and shape	Rod-shaped^51^ Approx. 2–5 µm length and 0.5–1.5 µm diameter^51^	Rod-shaped^52^ Approx. 0.5–2.0 µm length and 0.5–4 μm diameter^52^	Morphology and structure varying, depending on cell cycle^53^ 3.5–8 µm length^53^
Lipids	6.3% total lipids in dry weight^42^	6–7% total lipids in dry weight^43^	5–15% total lipids in dry weight^44^
Cell wall	Relatively thin (< 10 nm)^45^ Main components: lipopolysaccharide, lipoprotein, small amount of peptidoglycan (< 10% of total cell well)^54^	Relatively thick (20–80 nm)^45^ Mainly composed of peptidoglycan (30–70% total cell wall)^54^	Around 70–100 nm thick^55^ Mainly composed of two polysaccharides: polymers of mannose covalently linked to peptides, or mannoproteins, and polymers of glucose, or glucans^56^

### Factor 1: Microorganism size

In general, RF heating uniformity is greatly affected by the shapes and sizes of materials^41^. The size of the microorganisms in the current study follows the order *L. monocytogenes* ≤ *S.* Typhimurium < *S. cerevisiae*. Consequently, the larger size of *S. cerevisiae* as compared to the two bacteria is a possible reason for the higher observed heating rates for the former. This could be due to the fact that larger microorganisms are easier to polarize under the influence of an electromagnetic field, at the given frequencies. However, the aforementioned trend is not followed at 3.0 ± 0.02 MHz in NaCl, where the heating rate for *S. cerevisiae* was lower than expected. Hence, there was a frequency-dependent interaction effect between microorganisms and salt in simple media (as discussed in more detail below). Moreover, the higher heating rates observed for *L. monocytogenes* as compared to *S*. Typhimurium did not fit in the proposed trend of increasing heating rate with increasing microorganism size. Therefore, there may be other explanations for the observed differences in heating behavior among the microorganisms.

### Factor 2: Microorganism lipid content

The lipid content per dry weight of the microorganisms used in this study follows the order *S.* Typhimurium (6.3%) ≤ *L. monocytogenes* (6–7%) < *S. cerevisiae* (5–15%)^42–44^. In the current study, the heating rates followed the order *S.* Typhimurium ≤ *L. monocytogenes* ≤ *S. cerevisiae* for all conditions, except for NaCl medium at 3.0 MHz, for which the heating rate of *S. cerevisiae* was not significantly different compared to *S*. Typhimurium. Thus, the high heating rate of *S. cerevisiae* compared to bacteria might be related to the high content of lipids in the cells, due to the influence that lipids can have on RF heating. On the contrary, the absence of differences in lipid content between the two bacteria might attribute the higher heating rate of *L. monocytogenes* to differences in the cell membranes of the two bacteria.

### Factor 3: Microorganism cell membrane

The thick layer of peptidoglycan present in Gram-positive bacteria, contrary to Gram-negative bacteria^45^, possibly also affects heating behavior. The higher heating rate of *L. monocytogenes* as compared to *S*. Typhimurium can be related to the content of protein and carbohydrates in that layer, as they can have an effect on RF heating^31^. Also, an increase in protein is known to cause an increase in the dielectric loss factor^46^. The dielectric loss factor is associated with the absorption mechanisms of electromagnetic energy^47^; a low dielectric loss factor signifies less energy absorption, leading to a poor heating in an electromagnetic field^48^. Therefore, an increase in the dielectric loss factor due to the increase of protein, as observed in the present study, might result in more heat dissipation. Consequently, the presence of the peptidoglycan layer in Gram-positive bacteria is a possible explanation for the higher observed heating rate for *L. monocytogenes* than for *S*. Typhimurium.

### Factor 4: Salt in the heating medium

**Figure 3:**
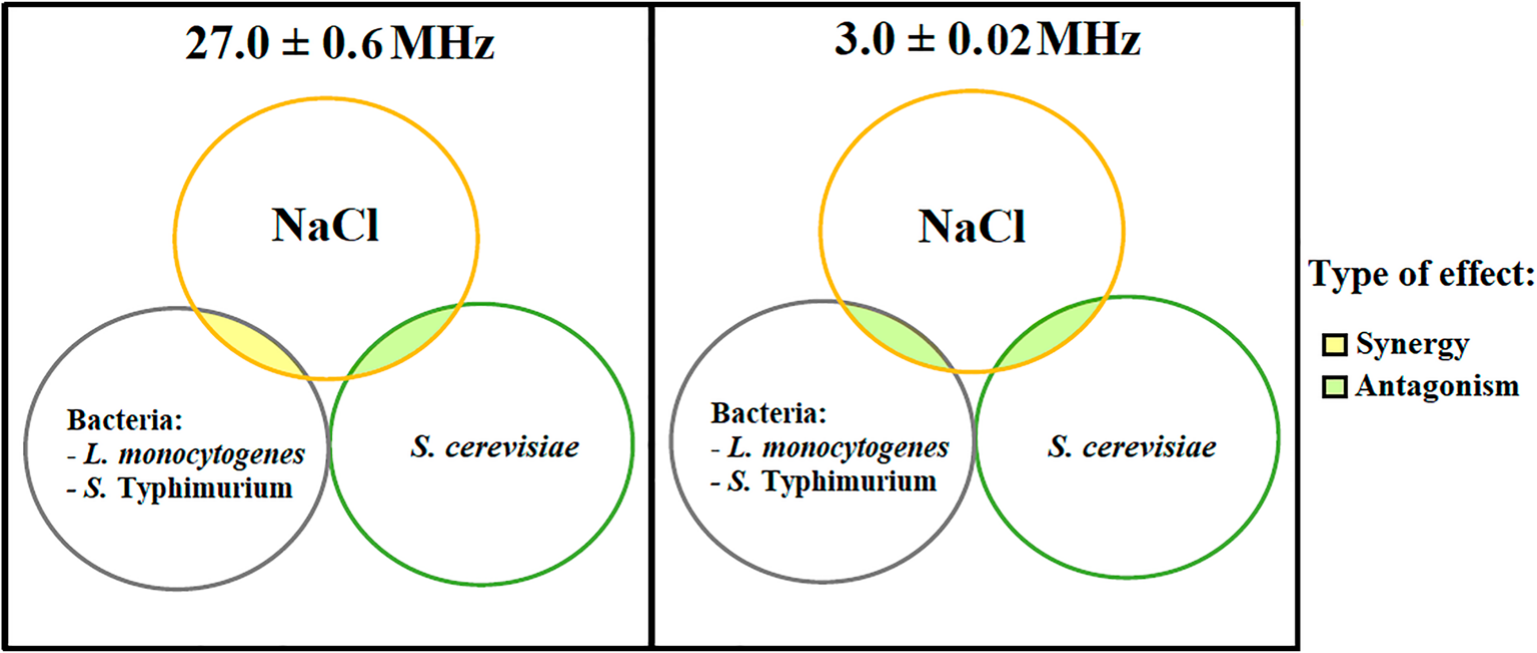
Interaction effect on RF heating of microorganisms and NaCl solution.

Apart from the effect of factors related to the cells of the microorganisms, salt in the heating medium also significantly affects RF heating. Figure 3 describes the interaction effect on RF heating between microorganisms and salt at both frequencies. Depending on the type of interaction, effects on heating can be defined as superposition (i.e., simple addition of temperature increase effects), synergy (i.e., the combined effect is larger than the sum of the separate two effects), and antagonism (i.e., the combined effect is smaller than the sum of the two separate effects). The presence of these effects was in the current study checked by conducting a separate statistical analysis (available in Supplementary Data S3 online). *S. cerevisiae* and salt exhibited an antagonistic effect for both frequencies. Bacteria and salt exhibited a synergistic effect at 27.0 ± 0.6 MHz, but an antagonistic effect at 3.0 ± 0.02 MHz. These different behaviors can possibly be linked to the cell morphology and composition of the three microorganisms, as discussed above.

The effect of salt has been already studied under RF treatment and its beneficial effect has been reported in different studies, such as uniformity of heating in beef^5^ as well as increasing the heating rate in RF pasteurization for pistachios^49^ at frequencies around 27 MHz. However, this does not apply to all frequencies, and it has also been reported that high salt content results in higher conduction losses at lower frequencies, which has a greater effect on the loss factor^50^. Although, dielectric properties of the heating media in the present study were not quantified, these phenomena could explain the observed lower heating rates at 3.0 ± 0.02 MHz than at 27.0 ± 0.6 MHz, as decreasing the frequency at a high NaCl content ends in less heat absorption. Furthermore, interaction of molecules and compounds in the media might affect the dielectric properties of the sample, e.g., depending on the size and charge of the dissolved ions, salt could bind free water molecules and lead to water polarization and subsequent decreases in the dielectric constant^47^. In this way, it is likely that there is an interaction between the composition of *S. cerevisiae* and the water molecules that cause a beneficial effect at 3.0 ± 0.02 MHz, counter-acting the increase in temperature caused by *S. cerevisiae*. However, there is no evidence of such effect in literature, meaning that more research towards this effect should be conducted, e.g., by conducting experiments at additional frequencies and salt concentrations.

## Conclusions

The isolated effect of microorganism morphology and composition on RF heating behavior in simple media was investigated. This study showcased a clear temperature-increasing effect of the presence of microorganisms during RF heating, although the extent of this effect was dependent on RF frequency. In general, the achieved heating rates followed the order *S*. Typhimurium ≤ *L. monocytogenes* ≤ *S. cerevisiae,* and the heating rates were higher at 27.5 ± 0.6 MHz than at 3.0 ± 0.02 MHz. The most likely explanations for this observed trend are related to the composition of the microorganisms, i.e., the high total cell lipid content of *S. cerevisiae*, on the one hand, and the high amount of peptidoglycan in *L. monocytogenes*, on the other. Nevertheless, some exceptions to this trend were observed, most likely due to interaction effects between microorganisms and salt. Especially the influence of yeast on RF heating behavior at different frequencies in the presence of salt is still not well described. Therefore, the isolated effect of yeast cells on RF treatments needs to be further investigated, possibly by using different RF frequencies, salt concentrations, and/or longer exposure times. Additionally, the effect of lower microbial concentration of both bacteria and yeasts on the RF heating behavior should also be studied, as microorganisms typically occur at significantly lower densities than the high cell densities employed in this study.

Moreover, it should be noted that during the short duration of the RF treatments, none of the microorganisms were inactivated at sublethal temperatures at any of the two frequencies. Treatment at the given frequencies hence did not result in nonthermal inactivation, although RF experiments at other frequencies should be performed in future studies in order to better understand the effect of RF on microorganisms.

In general, the current study was the first to investigate the effect of the presence of microorganisms on the heating behavior of simple media, setting the stage for possible future studies towards the design of more effective heating processes for the food and biotechnology industry.

## Supplementary Information

Supplementary Information 1.

Supplementary Information 2.

Supplementary Information 3.

## Data Availability

All data generated or analyzed during this study are included in this published article (and its supplementary information files).
